# Impact of Computational Histology AI Biomarkers on Clinical Management Decisions in Non-Muscle Invasive Bladder Cancer: A Multi-Center Real-World Study

**DOI:** 10.3390/cancers18020249

**Published:** 2026-01-14

**Authors:** Vignesh T. Packiam, Saum Ghodoussipour, Badrinath R. Konety, Hamed Ahmadi, Gautum Agarwal, Lesli A. Kiedrowski, Viswesh Krishna, Anirudh Joshi, Stephen B. Williams, Armine K. Smith

**Affiliations:** 1 Rutgers Cancer Institute, New Brunswick, NJ 08901, USA; 2 Allina Health, Minneapolis, MN 55407, USA; 3 Department of Urology, University of Minnesota, Minneapolis, MN 55455, USA; 4 Mercy Clinic, St. Louis, MO 63141, USA; 5 Valar Labs, Palo Alto, CA 94306, USA; lesli@valarlabs.com (L.A.K.); anirudh@valarlabs.com (A.J.); 6 Department of Surgery, Division of Urology, University of Texas Medical Branch, Galveston, TX 77555, USA; 7 Brady Urological Institute, Johns Hopkins University, Washington, DC 21218, USA

**Keywords:** non-muscle invasive bladder cancer, Artificial Intelligence, precision medicine, risk stratification, Bacillus Calmette–Guerin, Computational Histology, decision impact

## Abstract

**Background/Objectives**: Non-muscle invasive bladder cancer (NMIBC) management is increasingly complex due to conflicting guideline-based risk classifications, ongoing Bacillus Calmette–Guérin (BCG) shortages, and emerging alternative therapies. Computational Histology Artificial Intelligence (CHAI) tests are clinically available, providing insights from tumor specimens including predicting BCG responsiveness and individualized recurrence and progression risks, which may support precision medicine. This technology features biomarkers purpose-built for clinically unmet needs and has practical advantages including a fast turnaround time and no need for consumption of tissue or other specimens. We assessed the impact of such tests on physicians’ decision-making in routine, real-world NMIBC management. **Methods**: Physicians at six centers ordered CHAI tests (Vesta Bladder) at their discretion during routine NMIBC care. Tumor specimens were processed by a CLIA/CAP-accredited laboratory (Valar Labs, Houston, TX, USA) where H&E-stained slides were analyzed with the CHAI assay to extract histomorphic features of the tumor and microenvironment, which were algorithmically assessed to generate biomarker test results. For each case from 24 June 2024 to 18 July 2025, ordering physicians were surveyed to assess pre- and post-test management plans and post-test result usefulness. **Results**: Among 105 high-grade NMIBC cases with complete survey results available, primary management changed in 67% (70/105). Changes included modality shifts (n = 7; three to radical cystectomy with high prognostic risk scores; four avoiding cystectomy with low scores) and intravesical agent change (n = 63). Surveillance was intensified in 7%, predominantly among those with ≥90th percentile risk scores. The therapeutic agent changed in 80% (40/50) of predictive biomarker-present (indicative of poor response to BCG) tumors vs. 48% (23/48) of biomarker-absent tumors. **Conclusions**: In two thirds of cases, CHAI biomarker results influenced clinical decision-making during routine care. BCG predictive biomarker results frequently guided intravesical agent selection. These results have implications for optimizing clinical outcomes, especially in the setting of ongoing BCG shortages. Prognostic risk stratification results guided treatment escalation vs. de-escalation, including surveillance intensification and surgical vs. bladder-sparing decisions. CHAI biomarkers are currently utilized in routine clinical care and informing precision NMIBC management.

## Simple Summary

Non-muscle invasive bladder cancer is a common malignancy with a high rate of recurrence or progression. A supply shortage of the standard treatment and a wave of new treatment options have increased the need for precision medicine in this disease. Artificial intelligence-powered histology biomarkers have emerged as promising tools to support risk stratification and treatment selection in bladder cancer. We assessed the impact of this testing through routine care on physicians’ decisions for 105 patients and found that results influenced clinical decision-making in two thirds of cases, including changing therapeutic agent and intensifying treatment plans. These AI-powered tools are currently utilized in routine clinical care and can guide precision bladder cancer management.

## 1. Introduction

Non-muscle-invasive bladder cancer (NMIBC), which comprises approximately 75% of bladder cancer cases overall, arises from and remains confined to the urothelial lining and includes both papillary tumors (pathologic stages pTa and pT1) and carcinoma in situ (CIS; can be concurrent with papillary disease or isolated as pTis) [1,2]. Disease is biologically heterogeneous with a marked variance in natural history, with some lesions cured by surgical intervention alone, others with a high propensity for recurrence but low progression risk, and others with substantial risk of progression to muscle-invasive disease [3,4,5]. Standard management of NMIBC generally includes transurethral removal of the bladder tumor (TURBT) followed by surveillance or intravesical therapy with chemotherapy or Bacillus Calmette–Guérin (BCG); radical cystectomy may also be considered [2]. In recent years, management has grown increasingly complex due to an extended global supply shortage of BCG and a recent proliferation of emerging treatment options [6,7,8]. This ongoing shortage, combined with BCG’s variable efficacy—with up to 40% of patients recurring or progressing despite adequate treatment—has spurred increased interest in alternative intravesical treatments and more personalized care approaches [9,10,11,12].

Guidelines offer latitude to physicians to select from a wide range of acceptable treatment and management options for patients with NMIBC. Some guidance regarding management intensity is provided based on risk stratification. However, conflicting guideline-based risk classifications between organizations (e.g., American Urologic Association [AUA], European Association of Urology [EAU], International Bladder Cancer Group) can lead to varying risk-based recommendations for the same patient, and multiple options for guideline-concordant care exist even within risk strata [2,13,14,15]. Challenges with risk stratification and a bevy of treatment options without clear selection criteria make the clinical management of NMIBC a space in dire need of biomarkers to guide precision care.

While traditional clinicopathologic variables or genomic biomarkers have mixed performance in predicting treatment outcomes, advanced artificial intelligence-enabled biomarkers present a promising opportunity for precision medicine [16]. Some such tests are available for clinical use in some cancer types, including prostate cancer [17,18]. The Computational Histology Artificial Intelligence (CHAI) platform analyzes routinely available hematoxylin and eosin (H&E)-stained tumor specimens to extract features of the tumor and its microenvironment (including stroma and immune infiltrate), providing quantified data for features mapping to the hallmarks of cancer biology, including cell and nucleus size and shape, mitosis, apoptosis, and spatial architecture [19]. As H&E evaluation remains central to NMIBC diagnosis and reveals morphologic detail that is typically unexploited, deriving biomarkers directly from these existing resources enhances their utility without consuming additional tissue or altering standard workflows. The CHAI platform can then leverage deep learning to build algorithmic assessments of these features, which yield personalized biomarker results for individual patients.

CHAI biomarkers have been developed in multiple cancer types including pancreatic ductal carcinoma [19] and more recently NMIBC, including a predictive biomarker indicating likelihood of developing BCG-unresponsive disease and prognostic biomarkers providing risk scores for high-grade recurrence and muscle-invasive progression. These biomarkers have been validated in several multi-institution studies, in which they have been shown to prognosticate NMIBC outcomes independent of standard clinicopathological factors and predict relative treatment benefit between BCG and alternative therapies [20,21,22,23]. This evidence suggests that incorporating CHAI biomarker assessment could enhance precision in NMIBC care by identifying patients at higher risk for failure on BCG and those at greater risk of progression or recurrence who might benefit from treatment escalation. These tests are currently in clinical use in the US.

While prior studies established the prognostic and predictive accuracy of CHAI biomarkers, their actual impact on real-world clinical decision-making has not yet been examined. We conducted a multi-center study to assess how commercially available CHAI biomarker test results influence treatment planning for NMIBC in routine clinical practice. We hypothesized that knowledge of a patient’s CHAI biomarker results would guide physicians’ management (e.g., selecting between BCG and alternative intravesical treatment, treatment escalation or de-escalation) in a majority of cases. Here we report the decision impact of CHAI biomarker testing in NMIBC across six urology centers from diverse practice environments, using pre- and post-test physician surveys to capture changes in clinical management attributable to information from these novel biomarker assessments.

## 2. Materials and Methods

We performed an observational study across six U.S. urology centers selected from early adopters of this clinically available technology based on interest in evaluating the impact of CHAI biomarker testing on NMIBC management decisions and including both community and academic centers, diverse in size and geography. From 24 June 2024 through 18 July 2025, physicians integrated CHAI assays (Vesta Bladder BCGPredict and Vesta Bladder Risk Stratify) into routine care for patients with high-grade NMIBC at their discretion. There were no protocol-specified selection criteria aside from pathologically confirmed high-grade NMIBC; each participating physician was free to order testing for any patient with high-grade NMIBC where they felt additional information might aid management decision-making.

Testing was performed in a centralized CLIA/CAP-accredited laboratory (Valar Labs, Houston, TX, USA). For each case, ≥1 representative tumor slide or FFPE block was submitted; slides were cut and stained with H&E as needed. Slides were digitized at 40× magnification and analyzed using the CHAI platform, as previously described [9]. In brief, the platform extracted quantitative histomorphologic features from the tumor and microenvironment, which were input into locked algorithmic model(s) based on test(s) ordered. Results were issued to the ordering physician via a secure portal: (1) BCGPredict biomarker—predictive signature reported in a binary fashion in which biomarker-present status is associated with increased risk of BCG unresponsive disease, and/or (2) Risk Stratify biomarkers—prognostic signatures associated with risks of high-grade recurrence and progression to muscle-invasive disease, reported as percentile-based scores (0–100) for each endpoint.

To capture the impact of test results on clinical decision-making, pre- and post-test surveys were utilized (see Supplementary Materials). The physician was asked to complete a brief pre-test survey indicating their initial management plan for each case, including consideration of surgical intervention, intravesical treatment, and surveillance schedule, assuming no additional information beyond standard available clinical data. After CHAI test results were returned, a mirrored post-test survey of the management plan was issued to the physician. The post-test survey also asked which biomarker results were utilized to guide decision-making. Responses were communicated via secure submission, with cases assigned a study identifier; free-text management plan responses were centrally coded. Clinical variables provided by clinicians as part of routine test order submission (sex, age, stage, focality) and reported outputs of CHAI testing (biomarker status and scores) were incorporated into the study dataset using the assigned identifiers, with no patient identifiers included in the analytic dataset. Cases were included in the analysis only if both pre- and post-test surveys were available.

The primary endpoint was the proportion of cases in which there was a post-test change in primary clinical management, defined as any difference in indicated primary treatment modality (surgical vs. intravesical) or intravesical agent in the post- vs. pre-test plan. Secondary objective analyses included the nature of changes made stratified by biomarker results, as well as the proportion of physician-reported use of biomarker results to guide decision-making. Descriptive statistics were used to summarize the findings. Given the descriptive methodology and sample size, no comparative hypothesis testing was performed between subgroups.

## 3. Results

A total of 105 NMIBC cases met the inclusion criteria with complete survey data (Table 1). The cohort was predominantly male (71%) with a median age of 75 years (range 39–90). All patients had high-grade urothelial carcinoma histology; clinical stages at time of testing included 10 (10%) with pTis, 41 (39%) with pTa, and 54 (51%) with pT1; 8 patients with papillary disease had concurrent carcinoma in situ (CIS, 8%). In total, 46 (44%) tumors were multifocal, 42 (40%) were unifocal, and focality was unknown in 17 (16%). BCGPredict predictive biomarker test results revealed biomarker-present (indicative of poorer response to BCG) status in 54 (51%) cases and biomarker-absent status in 51 (49%). The median recurrence risk score was 71 (IQR 39–88), and the median progression risk score was 56 (IQR 25–85) ( Supplementary Figure S1).

In analysis of the primary objective, a post-test change in primary clinical management was made in 70 (67%) cases. In seven of these cases, surgical versus intravesical modality changed: three cases in which radical cystectomy (RC) was elected after receipt of high progression and/or recurrence risk scores, and four cases in which RC was deferred in favor of intravesical therapy after receipt of low risk scores. For the additional 63 cases with altered primary clinical management, a change occurred in the choice of specific intravesical agent. While not included in the primary endpoint, data on surveillance changes were also analyzed, with post-test surveys for seven (7%) patients indicating a management plan adjusted to intensify surveillance imaging and/or biopsy protocols; six of these cases had progression and/or recurrence risk scores above the 90th percentile.

**Figure 1:**
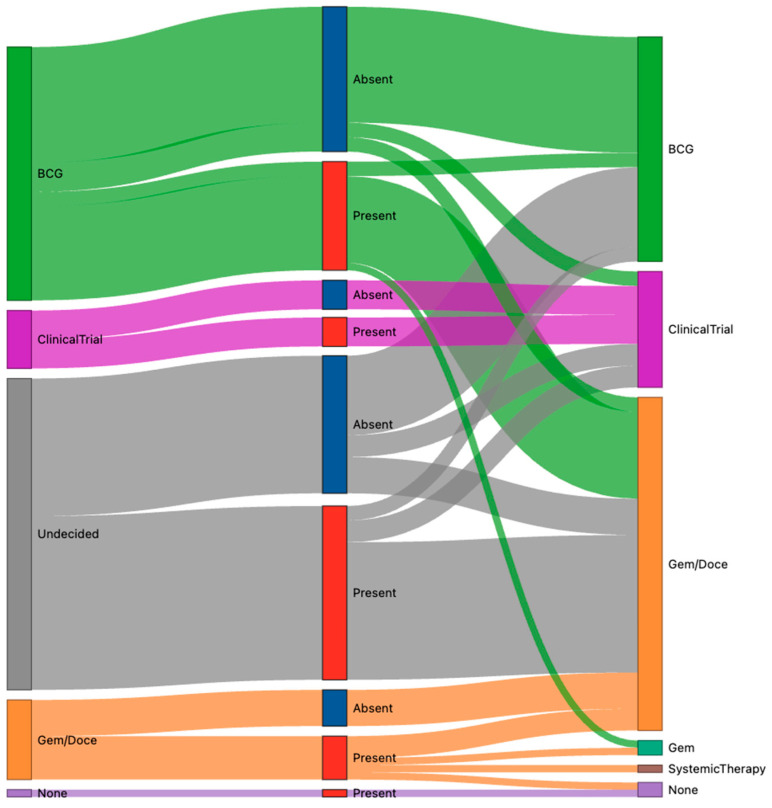
Pre- (left) versus post-test (right) intravesical therapy agent changes by Computational Histology AI (CHAI) BCG predictive biomarker status (center). BCG: Bacillus Calmette–Guérin. Gem/doce: gemcitabine/docetaxel. Gem: gemcitabine.

Excluding those patients with a primary modality change, pre- versus post-test intravesical therapy decisions for the remaining 98 cases were analyzed by predictive biomarker status (Figure 1). The intravesical agent decision changed after the receipt of test results in 40 of 50 (80%) biomarker-positive patients, with the most frequent being a post-test plan of intravesical chemotherapy (gemcitabine or gemcitabine + docetaxel [gem/doce]) from a pre-test plan of BCG (n = 13) or undecided intravesical agent (n = 19). Of the ten (20%) biomarker-positive patients with no change in their treatment plan, management included clinical trials (n = 4), gem/doce (n = 3), BCG (n = 2), and no treatment (n = 1).

Of the 48 patients in the intravesical agent analysis whose tumors were predictive biomarker-absent, 23 (48%) experienced an intravesical therapy change after testing, including 19 patients whose initial intravesical agent was undecided and changed post-testing to BCG (n = 11), gem/doce (n = 5), or a clinical trial (n = 3). Of the 25 (52%) of biomarker-absent patients whose intravesical treatment plan remained unchanged, the majority (n = 16) received BCG.

**Figure 2:**
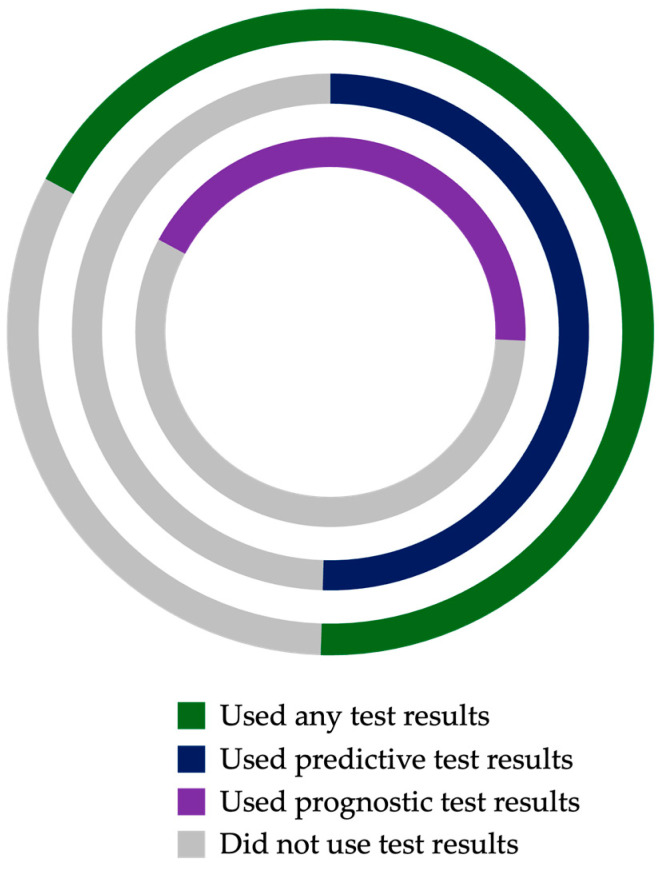
Physicians’ stated utilization of prognostic (inner), predictive (middle), and any (outer) Computational Histology Artificial Intelligence (CHAI) test results in clinical decision-making.

In addition to specific management plans, physicians’ stated use of CHAI biomarkers to guide clinical decision-making was assessed in the overall study cohort. Post-test responses indicated use of at least one biomarker test result in the majority (68%, n = 71) of cases. Of the cases for which biomarker results were utilized, physicians reported using both predictive and prognostic biomarker results in 27 (38%), only predictive biomarker results in 26 (37%), and only prognostic biomarker results in 18 (25%) (Figure 2).

## 4. Discussion

The treatment landscape of NMIBC is highly complex and rapidly evolving due to challenges in pathologic risk stratification, conflicting and shifting guidelines, suboptimal efficacy of therapeutics, ongoing BCG shortage, and an explosion of emerging novel therapies. In this setting, effective biomarkers to facilitate precision medicine and more tailored care are a significant unmet need [24]. We found that results of clinical AI-powered histology biomarker testing influenced real-world decision-making in the management of NMIBC. Following receipt of CHAI assay results, physicians altered their treatment plans in two thirds of cases, reflecting a substantial uptake of this tool to support decision-making in routine practice. This is the first study to assess the real-world clinical impact of these novel advanced biomarkers.

The most frequent management change was in the choice of intravesical agent: for BCG-specific predictive test results, biomarker-present patients predicted to have a poor response to BCG were more often recommended alternative therapies. Only 8% of patients whose pre-test intravesical agent was undecided ultimately received BCG, and of those for whom BCG was the original treatment plan but had biomarker-present test results, 87% were re-routed to intravesical chemotherapy. The majority of these patients ultimately received intravesical gemcitabine/docetaxel, a regimen for which randomized data is forthcoming but which has been shown to provide similar outcomes in unselected patients with NMIBC [25,26]. This regimen shift is supported by the recent study by Packiam et al. in which CHAI BCG predictive biomarker-positive patients experienced better outcomes when treated with gem/doce vs. with BCG, with high-grade recurrence-free survival at 2 years of 90% when receiving gem/doce compared to 56% when receiving BCG (HR 5.4, *p* = 0.007) [22]. In contrast, among biomarker-negative patients, there was no difference with BCG vs. gem/doce treatment. A predictive biomarker-selected approach will further optimize outcomes in intravesical treatment selection between these and other regimens. Of note, some predictive biomarker-present patients in our study ultimately received an intravesical agent through a clinical trial, in line with seeking an alternative therapy approach in a quickly evolving treatment landscape with many emerging novel agents for NMIBC and especially BCG-unresponsive disease [27].

Predictive biomarker-absent patients in this study were more often treated with BCG, with 69% of cases planning for BCG or an undecided agent ultimately treated with this standard therapy after receiving test results indicating an expected typical response to the therapy. However, 18% ultimately went on to receive gem/doce instead, reflective of both the increasing adoption of this regimen and treatment plan adaptation required due to the ongoing BCG shortage.

Physicians may use predictive biomarker testing to rationally allocate BCG across clinical practice in times of shortage. This selection process thus far has been largely arbitrary or based on logistic factors of availability and split dose scheduling, and biomarker-directed allocation is an improved method of responsible drug stewardship [28]. Pursuing alternative therapies in a biomarker-selected patient population less likely to benefit from BCG can not only optimize for improved clinical outcomes and avoid unnecessary toxicity from ineffective treatment in those patients but also allocate scarce medication to those more likely to benefit, given that biomarker-negative patients represent a subpopulation for whom BCG is justified and high-value despite shortages and other next-generation treatment options.

While less frequent, notable changes in management approach were also reported in this study based on prognostic test results. High-grade NMIBC is heterogeneous in outcomes, even within risk strata; some patients never recur after TURBT alone, while others progress rapidly even with intravesical therapy [29]. Traditional risk stratification tools, such as the EAU “high risk” vs. “very high risk” categorization or AUA risk tiers, are based on clinical features like tumor size, multifocality, and medical history. These models, however, have mixed performance and do not incorporate tumor biology [30]. The CHAI approach can leverage microscopic histologic patterns associated with aggressive biology to produce more granular risk estimates. A recent study by Chang et al. of 269 patients with high-grade pTa NMIBC found the CHAI prognostic biomarker outperformed both AUA and EAU risk models in predicting recurrence and progression, reclassifying 36–58% of patients’ risk groups [23].

In our study, this quantitative risk assessment of recurrence and progression likelihood via CHAI prognostic scores led to adjustments in surveillance regimens and surgical planning tailored to patients’ individual risk profiles. Several patients with high risk scores had plans for surveillance intensified, including earlier or more frequent imaging and/or biopsies, thresholds lowered for further evaluating potentially suspicious findings, and cystoscopy performed in the operating room and/or with blue light rather than in-office cystoscopy. Three patients ultimately underwent radical cystectomy following receipt of very high (≥90th percentile) risk scores, two of whom were found to have more extensive disease upon further workup. Of note, one patient originally planned to undergo intravesical therapy for their NMIBC diagnosis but ultimately received systemic chemotherapy due to progressive disease; this patient had both recurrence and progression scores ≥90th percentile. Radical surgery was also avoided in four patients with low risk scores in favor of bladder-sparing approaches deemed likely safe.

These management changes demonstrate the opportunity for physicians to utilize such test results to create more personalized approaches to NMIBC care: for instance, rather than subjecting all “high-risk” patients to uniform intense surveillance or pursuing cystectomy based on broad clinical risk group alone, patient counseling and shared decision-making can be tailored to more precise individual risks. Avoiding cystectomy in even a small number of patients is significant, given the morbidity and quality-of-life impact of such a procedure, and CHAI prognostication has potential to reduce the unnecessary surgery [31]. Conversely, identifying a patient with extreme risk of progression to invasive disease in order to expedite definitive therapy could be life-saving [32].

One finding of interest is the prevalence of BCG-predictive biomarker positivity in this cohort, with 51% of included cases demonstrating the presence of the biomarker for poor response to BCG compared to rates of approximately 30% biomarker positivity in previously published studies [20,22]. One possible explanation for this may be different treatment histories; whereas previous validation studies comprised BCG-naïve cases, this real-world cohort was selected at physicians’ discretion and may include patients with recurrent disease who were previously treated with BCG and could be enriched for biologic resistance. This increase in biomarker positivity was not observed in a published pilot study of BCG-treated NMIBC, which reported a biomarker presence rate of 19%, but this series was small (n = 52) [21]. As treatment history was not a required clinical variable for test order submission and thus was not available for inclusion in this analysis, the association of biomarker status with past treatment cannot be assessed here, but additional exploration of biomarker positivity rates in future studies will be important to better understand this potential trend.

Our findings must be interpreted in the context of the study design. First, this analysis was focused on test results and there is a possibility that management changes may not have been attributed to those results alone in every case; it is likely that some care decisions were also influenced by patient preference or other factors. However, physicians reported using CHAI biomarker test results to guide decision-making in 68% of cases. Of these, physicians indicated a near-even split of using predictive, prognostic, or both test results to guide management, in line with the paradigm of NMIBC care in which some clinical situations require escalation vs. de-escalation decisions, some require granular treatment selection, and some require decision support for both. It is anticipated that biomarker test results would be incorporated into multifactorial decision-making in a complementary fashion alongside other clinical and patient factors. Additionally, as NMIBC therapy continues to expand with multiple new FDA-approved agents and divergent guideline recommendations, the importance of validated, real-world biomarkers will only increase.

An additional limitation is that this study did not evaluate oncologic outcomes, which will be a focus of future research. Additionally, while our study spanned multiple centers, geographies, and practice environments, distribution of included patients was not equal, with two centers contributing more than 30 patients each and the remaining four centers each contributing fewer than 15 cases. At all centers, participating physicians were early adopters of this technology, and decisions regarding which patients to test were at physician discretion, which may introduce selection bias.

These limitations could be addressed by additional future studies incorporating more in-depth assessment of additional factors influencing clinical decision changes and including a broader range of participating sites to explore impacts in general urology practice and to identify any barriers to implementation. Nonetheless, our multi-institution experience demonstrates that CHAI biomarker testing is feasible in routine care—with a rapid turnaround, no need for special tissue handling or specimen consumption, and seamless integration via a digital portal—and that it adds clinically meaningful information to guide patient management.

Finally, it is worth noting that the paradigm of AI-powered pathologic analysis as a clinical decision tool is still emerging. This study provides a first glimpse into how such technology can directly affect therapeutic decision-making in oncology. As adoption of these tools becomes more widespread and additional AI-driven biomarkers become available—for example, in response to the plethora of emerging NMIBC treatments and in other oncologic disease states—continued study of the benefits of integrating such tools into clinical care will be key. The ultimate value of any biomarker lies not just in its performance but in whether it can be feasibly implemented and change clinical management to ultimately benefit patients. Here we show that use of CHAI biomarkers did impact management plans, supporting more personalized and evidence-aligned care for patients with NMIBC and aligning with broader trends in oncology toward individualized treatment selection and precision medicine. In an era defined by therapeutic expansion, guideline divergence, persistent BCG shortages, and unmet need for precision selection of intravesical and systemic therapies, these data provide compelling evidence that AI-driven biomarkers can play a pivotal role in guiding personalized NMIBC management.

## 5. Conclusions

In this multi-center real-world study, integration of a CHAI biomarker test using H&E pathology specimens into routine NMIBC care meaningfully impacted clinical decision-making. Two thirds of cases had changes in management following receipt of test results. The CHAI predictive biomarker for BCG unresponsiveness enabled more rational intravesical therapy choices, directing patients less likely to benefit from BCG toward alternative treatments and promoting rational allocation of scarce therapy. Prognostic biomarker risk scores allowed tailoring of surveillance approaches and more precise decision-making about bladder-sparing treatment versus early cystectomy. While further studies are needed to assess implementation in broader settings and impact on oncologic outcomes, this work provides early evidence of the practical clinical utility of AI-powered histology assays in delivering clinically actionable guidance that enhances precision medicine for bladder cancer. In an era of expanding NMIBC treatment options, rising utilization of newer intravesical agents and ongoing BCG shortages, AI-driven decision support via CHAI biomarker assessment fills a critically unmet need for precision medicine in navigating this increasingly complex therapeutic landscape. Ultimately, routine use of these biomarkers may improve patient outcomes, reduce unnecessary interventions, and optimize resource utilization, an important step toward truly personalized care in bladder cancer.

## Figures and Tables

**Table 1:** Cohort characteristics (n = 105).

Characteristic		n	%
Sex	Male	75	71
Age (Median, Range)		75 (39–90)	
Stage	pTis	10	10
	pTa	41	39
	pT1	54	51
Concurrent CIS		8	8 *
Focality	Multifocal	46	44
	Unifocal	42	40
	Unknown	17	16
CHAI BCG Predictive Biomarker Status	Present	54	51
	Absent	51	49
CHAI Risk Biomarker Scores (Median, IQR)	Recurrence	71 (39–88)	
	Progression	56 (25–85)	

CIS: carcinoma in situ. CHAI: Computational Histology Artificial Intelligence. BCG: Bacillus Calmette–Guérin. IQR: interquartile range. * Percentage of papillary cases.

## Data Availability

Data in this study may be requested from the corresponding author, contingent upon a data use agreement with qualified investigators and an assessment of privacy risk for contributing clinicians.

## Supplementary Materials

The following supporting information can be downloaded at: https://www.mdpi.com/article/10.3390/cancers18020249/s1, Figure S1. Scatterplot of Computational Histology Artificial Intelligence (CHAI) reported recurrence and progression risk scores in each case.

## Abbreviations

The following abbreviations are used in this manuscript:

NMIBC	Non-muscle invasive bladder cancer
BCG	Bacillus Calmette–Guérin
CHAI	Computational Histology Artificial Intelligence
AUA	American Urologic Association
EAU	European Association of Urology
H&E	Hematoxylin & eosin
CIS	Carcinoma in situ
RC	Radical cystectomy
Gem/doce	Gemcitabine + docetaxel
