# Supplementary material for: Impact of Computational Histology AI Biomarkers on Clinical Management Decisions in Non-Muscle Invasive Bladder Cancer: A Multi-Center Real-World Study
Source: Cancers (Basel). 2026 Jan 14;18(2):249. doi: 10.3390/cancers18020249 (PMC12839055; doi:10.3390/cancers18020249)
Supplement: Supplementary file 1 [file cancers-18-00249-s001.zip › cancers-4083506-supplementary.pdf]

Supplementary Information

Presented results analyzed the following survey questionnaire items:
Pre-test questionnaire items

- Are you considering cystectomy?
- Are you considering intravesical treatment? If so, which one?
- What is the maintenance/surveillance schedule you are considering?

Post-test questionnaire items

- Are you considering cystectomy?
- Are you considering intravesical treatment? If so, which one?
- What is the maintenance/surveillance schedule you are considering?
- Any other management changes outside those asked?
- Which part(s) of the results (biomarker group, recurrence score, progression score) did you use?

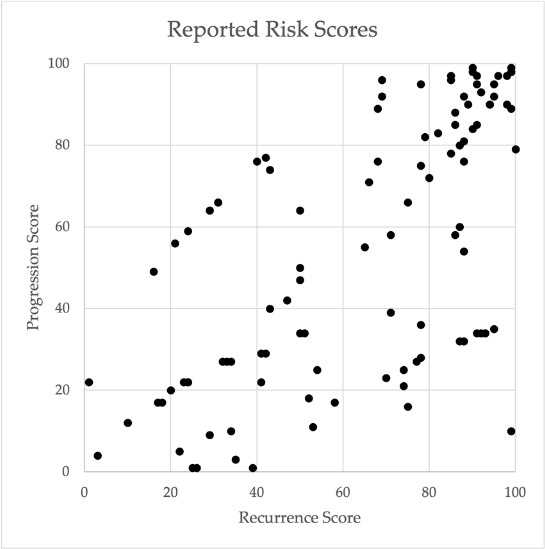

Supplementary Figure S1. Scatterplot of Computational Histology Artificial Intelligence (CHAI) reported recurrence and progression risk scores in each case.
